# The utility of syndromic respiratory pathogen panels: the premise of flexible and customizable approaches

**DOI:** 10.1128/jcm.00313-25

**Published:** 2025-06-10

**Authors:** Julie M. Norton, Gaby Dashler, Eili Klein, Heba H. Mostafa

**Affiliations:** 1 Department of Pathology, Division of Medical Microbiology, Johns Hopkins School of Medicine 1500 , Baltimore, Maryland, USA; 2 Department of Emergency Medicine, Johns Hopkins School of Medicine 1500 , Baltimore, Maryland, USA; 3 Center for Disease Dynamics, Economics, and Policy 439584, Washington, DC, USA; St Jude Children's Research Hospital, Memphis, Tennessee, USA

**Keywords:** syndromic panels, respiratory panels, respiratory viral testing

## Abstract

Extended respiratory panels have been limited to specific patient populations due to cost and inconclusive clinical utility. Customizing syndromic panels offers a way to balance clinical utility and available resources. In this study, we evaluated strategies and assessed the value of flexible, customized respiratory panels. A total of 200 specimens from symptomatic patients (December 2023 to September 2024), negative for SARS-CoV-2/Flu/RSV, were tested with the LIAISON PLEX Respiratory Flex Assay—an extended respiratory panel that offers flexibility in target selection. The study assessed additional diagnoses, correlations with institutional and state-wide pathogen prevalence, and whether customizable panels could optimize diagnostic yield. Sixty-two samples (31%) negative for SARS-CoV-2/Flu/RSV tested positive for other targets, primarily rhinovirus/enterovirus (60%), correlating with local and state prevalence. Weighted estimates for 18,373 symptomatic patients during the study period modeled a prevalence of 14.3% for rhinovirus/enterovirus, followed by HPIV-3, adenovirus, and coronavirus. During the study period, 6% of patients received the standard of care extended respiratory panel order after a negative SARS-CoV-2/Flu/RSV result, duplicating SARS-CoV-2/Flu/RSV testing. Leveraging a flexible feature could have resulted in an estimated staff time reduction of 5,545 minutes for a second swab collection and running a second test, in addition to the cost of running two different panels during a single encounter. Local respiratory pathogen prevalence data can guide target selection in customized panels. The inclusion of high-prevalence targets can increase the likelihood of diagnosis from 12% to nearly 30%. Flexibility in customizing targeted pathogen panels could enhance diagnostic value while conserving institutional resources.

**IMPORTANCE:**

Rapid and accurate identification of pathogens causing respiratory tract infections can aid in guiding treatment decisions, reducing healthcare costs, and supporting real-time surveillance of infectious diseases within a community. Limitations of clinical utility beyond SARS-CoV-2/Flu/RSV are primarily driven by cost and the lack of specific treatment options. There is a need to balance clinical gaps with testing cost and diagnostic stewardship. In this study, we evaluated the utility of flexible, customized respiratory viral panels and reportable targets within a broader set of available targets in an extended respiratory panel.

## INTRODUCTION

Syndromic respiratory panels are molecular-based diagnostics that detect and differentiate between respiratory pathogens responsible for respiratory tract infections. Panels of 3–5 targets (e.g., SARS-CoV-2/Flu/RSV) are more commonly used, particularly during the respiratory or influenza season (1). Large panels of 12 or more targets have become a standard of care for specific patient populations, including immunocompromised individuals (2). Current procedure terminology (CPT) codes that are used by Medicare, Medicaid, and other insurers for describing healthcare procedures differ based on the size of the panel, with different CPT codes for panels of less than 5, 6–11, and 12–25 + targets. The high cost and reduced likelihood of reimbursing extended respiratory panels (more than five targets) limited their use, particularly when a defined clinical utility is lacking.

In the United States, data from the most recent College of American Pathologists Infectious Disease respiratory survey indicate that the most commonly utilized extended respiratory panels are the BioFire panels (bioMérieux). Additional panels from Roche (ePlex), Luminex, and Qiagen have been adopted in clinical practice (3). These panels are multiplexed qualitative assays designed to detect and differentiate approximately 19 bacterial and viral nucleic acids, including influenza A, influenza A H1, influenza A H3, influenza B, respiratory syncytial virus (RSV A and B), parainfluenza (PIV1–4), adenovirus, coronavirus (229E, HKU1, NL63, and OC43), coronavirus SARS-CoV-2, human metapneumovirus, rhinovirus/enterovirus, *Chlamydia pneumoniae*, and *Mycoplasma pneumoniae*. The LIAISON PLEX Respiratory *Flex* Assay (Diasorin) received 510(k) clearance from the U.S. Food and Drug Administration (FDA) in March 2024. The LIAISON PLEX panel detects and differentiates similar viral and bacterial pathogens as preceding panels. However, the *Flex* software allows for target customization and the creation of smaller panels.

Given the increased cost associated with extended syndromic panels, customizing smaller panels based on target prevalence, institutional guidelines, and clinical needs may help reduce costs and conserve resources. In this study, we used the LIAISON PLEX Respiratory *Flex* Assay as a use case to assess the clinical relevance of small vs large panel testing in diagnosis and patient management. A cohort of remnant samples collected from symptomatic patients who tested negative for SARS-CoV-2/Flu/RSV was tested with the LIAISON PLEX Respiratory *Flex* Assay. Viral pathogens associated with symptoms were identified. Institutional cumulative clinical data, along with pathogen prevalence data, were used to evaluate the utility of extended panels and the likelihood of missing diagnoses when extended testing is not performed.

## MATERIALS AND METHODS

### Study population

Symptomatic patients presenting to the Johns Hopkins Hospital, Bayview Medical Center, or Johns Hopkins Outpatient Center between 01 December 2023 and 30 September 2024 and who had nasopharyngeal swabs collected and tested in the Johns Hopkins Medical Labs following the standard of care diagnosis (using the GeneXpert or the GenMark ePlex respiratory pathogen panels (RP2) [4, 5]) were included. The GeneXpert panel targets include SARS-CoV-2, influenza A and B, and RSV. The GenMark ePlex respiratory pathogen panel targets include SARS-CoV-2, adenovirus, coronavirus (229E, HKU1, NL63, and OC43), SARS-CoV-2, human metapneumovirus, human rhinovirus/enterovirus, influenza A, influenza A H1, influenza A H1-2009, influenza A H3, influenza B, parainfluenza virus 1, parainfluenza virus 2, parainfluenza virus 3, parainfluenza virus 4, RSV A, RSV B, *Chlamydia pneumoniae*, and *Mycoplasma pneumoniae*. Patient chief complaints and type of test ordered (e.g., excluded asymptomatic testing) were used to define symptomatic patients. A random set of samples (50 negative and 51 positive), previously tested with the standard of care panels, stratified by age and gender, was collected for validation of the LIAISON PLEX Respiratory *Flex* Assay. The LIAISON PLEX Respiratory Flex Assay targets are similar to the ePlex in addition to *Bordetella holmesii, Bordetella parapertussis*, and *Bordetella pertussis*. Additionally, 200 SARS-CoV-2/Flu/RSV-negative samples from symptomatic individuals who were not tested by the GenMark ePlex respiratory pathogen panels were selected for testing with the LIAISON PLEX Respiratory *Flex* Assay. Notably, the study was retrospective, and the LIAISON PLEX Respiratory *Flex* Assay results were not reported to patients or providers.

### Clinical data

Clinical data were bulk extracted from the electronic medical record. Demographic variables, such as age and gender, were collected alongside clinical characteristics, including chief complaints and respiratory testing results. Descriptive statistics summarizing demographic and clinical characteristics, as well as data analyses, were conducted using Stata version 18.0.

### Statistical analysis

We employed iterative proportional fitting, adjusting for age category and sex to estimate the positivity rates of respiratory pathogens other than SARS-CoV-2/Flu/RSV in all symptomatic patients who were negative for SARS-CoV-2/Flu/RSV between December 2023 and September 2024. The 200 clinical samples tested with the LIAISON PLEX Respiratory *Flex* Assay provided positivity rates for each pathogen that were used for weighted analyses. We then iteratively adjusted the weights of the clinical sample such that the weighted distribution of covariates matched the marginal distributions of the larger population. This process was performed separately for each pathogen to account for potential differences in the relationships between covariates and pathogen positivity, thereby reducing bias and improving the accuracy in comparisons. Weighted estimates of positivity rates for each respiratory pathogen were then calculated. All analyses were done in Stata version 18.0.

## RESULTS

### Concordance of the LIAISON PLEX Respiratory *Flex* Assay with the standard of care ePlex RP2

A total of 50 negative and 51 positive samples tested with the ePlex RP2 panel were tested with the LIAISON PLEX Respiratory *Flex* Assay. Sample collection dates were from 20 November 2023 to 31 July 2024. Positive samples tested included adenovirus (3), *Chlamydia pneumoniae* (1), coronavirus (4), SARS-CoV-2 (5), influenza A (3), influenza B (4), human metapneumovirus (4), *Mycoplasma pneumoniae* (4), HPIV1 (3), HPIV2 (4), HPIV3 (3), HPIV4 (4), rhinovirus/enterovirus (3), RSV-A (4), and RSV-B (3) (Table S1). When compared to the ePlex RP2, the LIAISON PLEX Respiratory *Flex* Assay showed 90% positive percent agreement (95% confidence interval [CI] 78.19%–96.67%) and 98% negative percent agreement (95% CI 89.35%–99.95%; Table 1). Five positive samples by the ePlex RP2 (two SARS-CoV-2; and one each human metapneumovirus, coronavirus, and HPIV4) tested negative by the LIAISON PLEX Respiratory *Flex* Assay and one negative sample by the ePlex RP2 tested positive for *Mycoplasma pneumonia*e by the LIAISON PLEX Respiratory *Flex* Assay (Table S1). Discordant analysis using the BioFire RP.2 test confirmed three of the six discordant results (Table S1).

**TABLE 1:** Concordance between the LIAISON PLEX Respiratory *Flex* Assay and the ePlex RP2 panel^*a*^

	ePlex RP2 (standard of care)	
LIAISON PLEX Respiratory *Flex*	Positive	Negative
Positive	45	1
Negative	5	49

^*a*^ Of the 51 positive samples tested, one sample was invalid and was not included in the agreement analysis.

### Target positivity rates during the clinical study time frame

**Fig 1:**
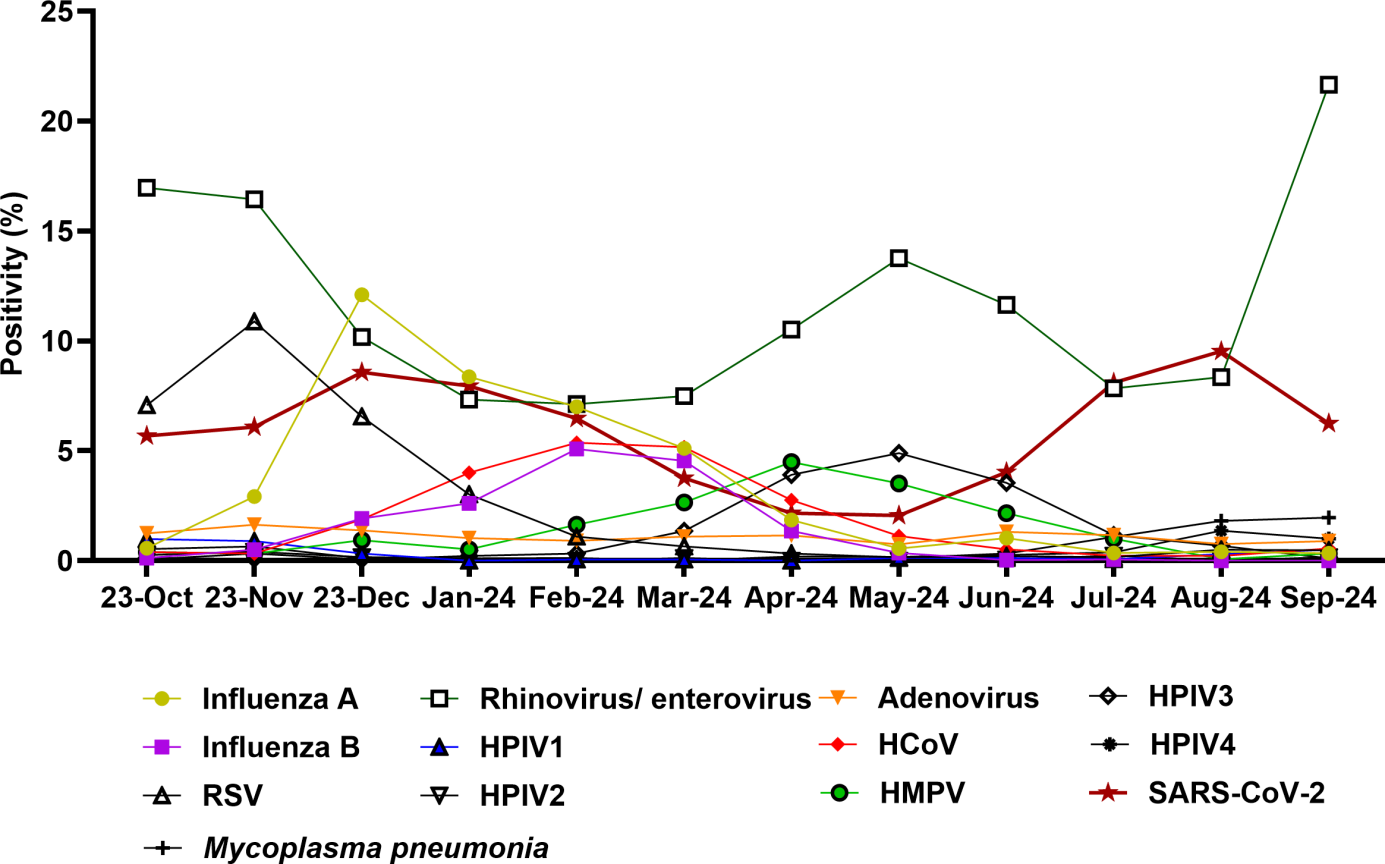
Positivity rates, October 2023 to September 2024. Shown is the positivity of the standard of care tests that include the GeneXpert and the ePlex RP.2 panels. HMPV, human metapneumovirus; HCoV, human coronavirus; HPIV, human parainfluenza virus.

During the clinical study time frame (December 2023 to September 2024, total tested samples with ePlex ~15,684), samples from patients who tested negative for SARS-CoV-2/Flu/RSV were collected for testing with the LIAISON PLEX Respiratory *Flex* Assay. During this time frame, cumulative laboratory positivity data showed that the highest average positivity was for rhinovirus/enterovirus (10.6%), followed by SARS-CoV-2 (5.9%) and influenza A (3.7%). Seasonal trends were notable (Fig. 1), with rhinovirus/enterovirus peaking in September 2024 (21.6%), influenza A peaking in December at 12.1% positivity, and SARS-CoV-2 highest positivity noted in August 2024 (9.5%). Increased circulation of other respiratory pathogens was observed, including influenza B (peak in February 2024 of 5.1%), HPIV3 (4.9% in May 2024), human metapneumovirus (4.5% in April 2024), and unusually increased circulation of *Mycoplasma pneumonia* (2% positivity in September 2024). Our data were consistent with Maryland State positivity data (6).

### Pathogens identified by the LIAISON PLEX Respiratory *Flex* Assay during the clinical study time frame

**Fig 2:**
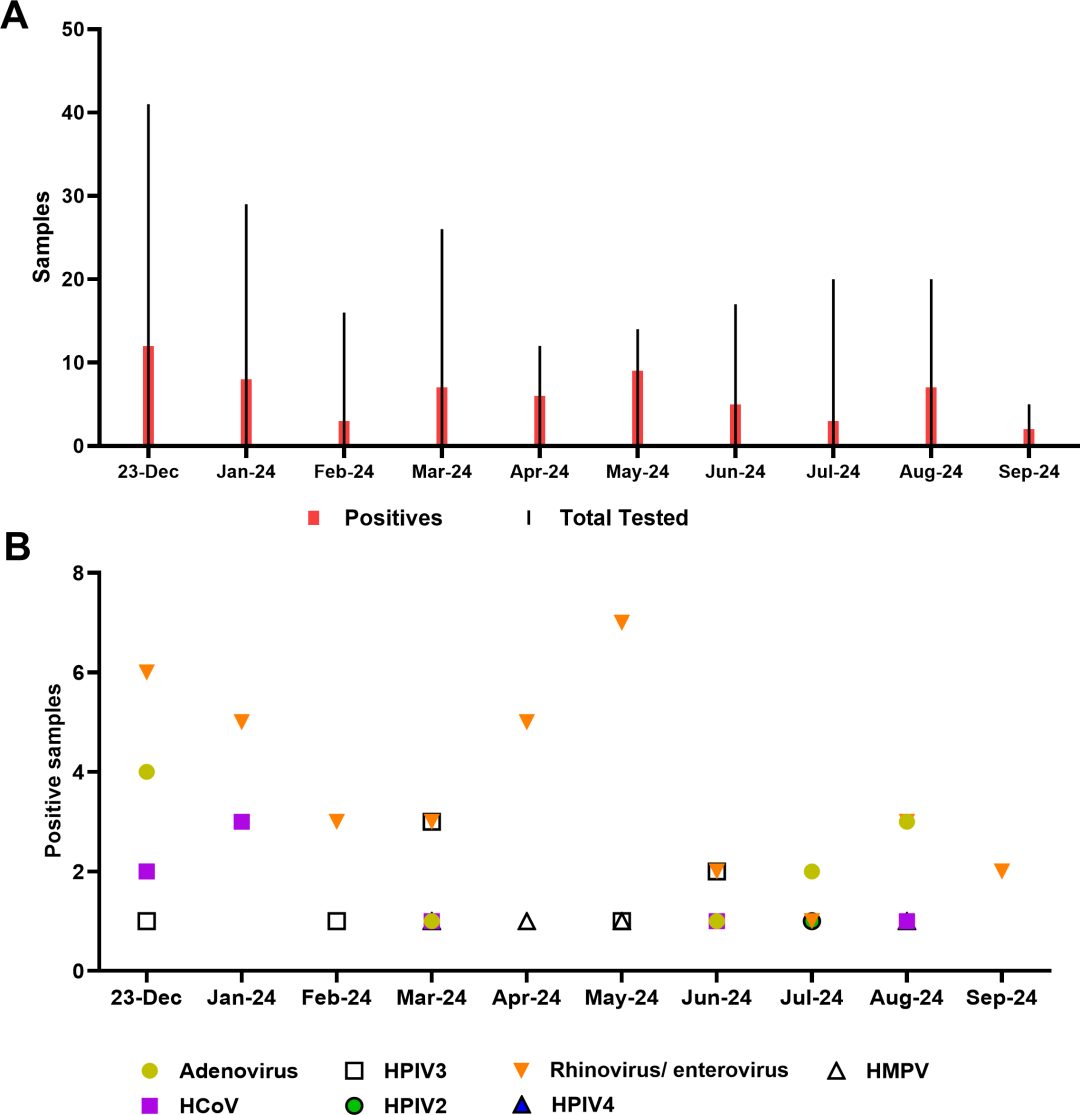
Pathogens identified by the LIAISON PLEX Respiratory *Flex* Assay. (**A**) Total tests and positives identified each month. (**B**) Targets identified each month of the study period. HMPV, human metapneumovirus; HCoV, human coronavirus; HPIV, human parainfluenza virus.

Of 200 samples collected from symptomatic individuals between December 2023 and September 2024, that tested negative for SARS-CoV-2/Flu/RSV, a total of 62 tested positive by the LIAISON PLEX Respiratory *Flex* Assay (31%). Positives were identified monthly during the study time frame regardless of the total number tested (Table 2). The majority of samples were positive for rhinovirus/enterovirus (37 samples, 60%), followed by adenovirus (18%), coronavirus, and HPIV3 (13% each; Table 2; Fig. 2). This correlated with viral positivity rates, particularly rhinovirus/enterovirus (Fig. 1) and the year-long circulation of adenovirus. The detection of coronavirus and human metapneumovirus was primarily during the time frame of their increased circulation (Table 2).

**TABLE 2:** Samples tested with and targets detected by the LIAISON PLEX Respiratory *Flex* Assay during the study time frame^*a*^

	Dec-23	Jan-24	Feb-24	Mar-24	Apr-24	May-24	June-24	July-24	Aug-24	Sep-24	Total	Coinfections	Target average positivity, JHHS data
Total tested	41	29	16	26	12	14	17	20	20	5	200		
Positives	12	8	3	7	6	9	5	3	7	2	62	7	
Detected targets													
Rhinovirus/enterovirus	6	5	3	3	5	7	2	1	3	2	37		10.59
Adenovirus	4			1			1	2	3		11	4	1.05
Coronavirus	2	3		1			1		1		8	4	2.19
Human metapneumovirus				1	1	1					3	4	1.73
Human parainfluenza virus 2								1			1		0.09
Human parainfluenza virus 3	1		1	3		1	2				8	3	1.63
Human parainfluenza virus 4									1		1		0.38

^*a*^ Highlighted are months with positivity above the viral target average per cumulative data from the Johns Hopkins Health System (JHHS), which is shown in the far right as a reference.

### Clinical characteristics of the study cohort

The overall median age of the 200-patient study cohort was 28.5 years (Interquartile Range [IQR]: 6–54), with more than half of the patients identifying as Black and females accounting for 52% (Table 3). The most frequently reported comorbidities were hypertension (28.5%), lung disease (24.5%), and immunosuppression (22.5%). Common presenting symptoms included flu-like symptoms (16.5%), shortness of breath (16.5%), and fever (13.5%). Regarding patient disposition, 16% were admitted to the hospital, while 58% were discharged from the emergency department (ED). The most frequently documented ED diagnoses were viral illness (11%), shortness of breath (7%), and upper respiratory infection (6.5%; Table 3).

**TABLE 3:** Socio-demographic and clinical characteristics among 200 symptomatic patients tested by LIASISON PLEX Respiratory *Flex* Assay

Characteristics	Category	Total	Positive	Negative
Overall		200	62	138
A. Socio-demographics				
Age (years)	Mean	31.3 ± 26.1	11.4 ± 15.5	40.2 ± 25.0
	Median	28.5 (6 - 54)	5 (1 - 12)	42 (17 - 62)
Sex	Female	104 (52.0)	34 (54.8)	70 (50.7)
	Male	96 (48.0)	28 (45.2)	68 (49.3)
Race	Black or African American	119 (59.5)	37 (59.7)	82 (59.4)
	White	46 (23.0)	15 (24.2)	31 (22.5)
	Other Race	35 (17.5)	10 (16.1)	25 (18.1)
Ethnicity	Not Hispanic or Latino	131 (65.5)	38 (61.3)	93 (67.4)
	Hispanic or Latino	12 (6.0)	2 (3.2)	10 (7.2)
	Not specified	57 (28.5)	22 (35.5)	35 (25.4)
B. Clinical characteristics				
Comorbidities	Lung disease	49 (24.5)	14 (22.6)	35 (25.4)
	Kidney disease	29 (14.5)	1 (1.6)	28 (20.3)
	Immunosuppression	45 (22.5)	6 (9.7)	39 (28.3)
	Diabetes	30 (15.0)	4 (6.5)	26 (18.8)
	Heart failure	27 (13.5)	2 (3.2)	25 (18.1)
	Hypertension	57 (28.5)	3 (4.8)	54 (39.1)
	Smoker	36 (18.0)	3 (4.6)	33 (24.4)
	Cancer	47 (23.5)	4 (6.5)	43 (31.2)
	Coronary artery disease	38 (19.0)	2 (3.2)	36 (26.1)
Chief complaint	Cough	13 (6.5)	6 (9.7)	7 (5.1)
	Emesis	18 (9.0)	7 (11.3)	11 (8.0)
	Fever	27 (13.5)	15 (24.2)	12 (8.7)
	Flu-like symptoms	33 (16.5)	12 (19.4)	16 (11.6)
	Sore throat	16 (8.0)	6 (9.7)	10 (7.2)
	Shortness of breath	33 (16.5)	8 (12.9)	25 (18.1)
Disposition status	Admitted	32 (16.0)	4 (6.5)	28 (20.3)
	Hospitalized observation	24 (12.0)	3 (4.8)	21 (15.2)
	Discharged	116 (58.0)	50 (80.7)	66 (47.8)
	Other	28 (14.0)	5 (8.1)	23 (16.7)
ED diagnosis	Asthma	7 (3.5)	4 (6.5)	3 (2.2)
	Cough	5 (2.5)	2 (3.2)	3 (2.2)
	Emesis	7 (3.5)	2 (3.2)	5 (3.6)
	Fever	11 (5.5)	4 (6.5)	7 (5.1)
	Gastroenteritis	9 (4.5)	5 (8.1)	4 (2.9)
	Pneumonia	5 (2.5)	1 (1.6)	4 (2.9)
	Shortness of breath	14 (7.0)	5 (8.1)	9 (6.5)
	Sore throat	13 (6.5)	2 (3.2)	11 (8.0)
	Upper respiratory infection	13 (6.5)	8 (12.9)	5 (3.6)
	Viral illness	22 (11.0)	16 (25.8)	6 (4.3)
Antibiotics prescribed	Amoxicillin	3 (1.5)	2 (3.2)	1 (0.7)
	Amoxicillin-clavulanate	2 (1.0)	0 (0.0)	2 (1.5)
	Azithromycin	1 (0.5)	0 (0.0)	1 (0.7)
	Cefpodoxime	1 (0.5)	0 (0.0)	1 (0.7)
	Cephalexin	3 (1.5)	0 (0.0)	3 (2.2)
	Clindamycin	2 (1.0)	1 (1.6)	1 (0.7)
	Penicillin V	1 (0.5)	1 (1.6)	0 (0.0)
	Sulfamethoxazole/trimethoprim	1 (0.5)	0 (0.0)	1 (0.7)

Among the 62 patients who tested positive with the LIAISON PLEX Respiratory *Flex* Assay, the median age was notably lower at 5 years (IQR: 1–12). Lung disease was the most frequently reported comorbidity (22.6%), and the most common presenting symptoms were fever (24.2%) and flu-like symptoms (19.4%). Hospital admission was required for only 6.5% of these patients. The most frequently assigned ED diagnoses among positive cases were viral illness (25.8%) and upper respiratory infection (12.9%; Table 3). Notably, four patients received antibiotic prescriptions, only two of whom had positive group A Streptococcal throat swabs, and two received an antibiotic prescription for lobar pneumonia (and tested positive for rhinovirus/enterovirus with the LIAISON PLEX Respiratory *Flex* Assay) and acute otitis media (tested positive for HPIV3 with the LIAISON PLEX Respiratory *Flex* Assay).

In contrast, among the 138 patients who tested negative, the median age was significantly higher at 42 years (IQR: 17–62). This group had a higher prevalence of chronic conditions, with hypertension (39.1%), cancer (31.2%), and immunosuppression (28.3%) being the most reported comorbidities. Hospital admission was more frequent among negative cases, with 20.3% requiring inpatient care. The most documented ED diagnoses in this group included sore throat (8.0%), shortness of breath (6.5%), and fever (5.1%; Table 3).

### Positivity modeling to local and statewide positivity

Based on weighted estimates of positivity among all symptomatic patients who tested negative for SARS-CoV-2/Flu/RSV between December 2023 and September 2024, rhinovirus/enterovirus showed the highest positivity rate at 14.3% (95% CI: 6.9%–27.4%). This was followed by HPIV3 at 2.7% (95% CI: 1.4%–5.0%), adenovirus at 2.6% (95% CI: 1.4%–4.5%), coronavirus at 2.1% (95% CI: 1.0%–4.3%), and human metapneumovirus at 0.7% (95% CI: 0.2%–2.1%).

When compared to Maryland state-level data for the same period (6), rhinovirus/enterovirus also had the highest positivity rate at 14.2%, while HPIV3, adenovirus, and human metapneumovirus were detected at 0.9%, 2.3%, and 3.4%, respectively.

### Respiratory panel utilization after negative SARS-CoV-2/Flu/RSV

During the study period, 18,373 patients underwent testing with the GeneXpert Xpress SARS-CoV-2/Flu/RSV Plus assay. Among them, 1,109 (6%) also received the GenMark ePlex respiratory pathogen panel within 72 hours of the initial test order. For these patients, the time interval between the initial GeneXpert test and the subsequent ePlex test had a mean of 19.8 hours (SD: 16.6) and a median of 14.6 hours (IQR: 6.9–28.6). Estimates of using a flexible solution that allows adding on targets to a sample already tested for SARS-CoV-2/Flu/RSV include, at a minimum, 3 minutes to collect a second swab (3,327 minutes) and 2 minutes to run the ePlex panel (2,218 minutes), in addition to the cost associated with duplicate testing of SARS-CoV-2/Flu/RSV.

## DISCUSSION

Viral upper respiratory tract infections are among the most common healthcare problems and reasons for healthcare visits. Although most cases are self-limiting and do not require medical intervention, severe disease can occur, particularly in elderly, immunocompromised, and pediatric patients (1, 7–9). Available interventions are limited to antivirals for SARS-CoV-2 and influenza (10, 11), as well as vaccines currently recommended for influenza, SARS-CoV-2, and RSV (12–15). Seasonal and year-round disease can be caused by various virus groups beyond SARS-CoV-2, influenza, and RSV (1, 16). Symptoms often overlap, and the clinical diagnosis of viral respiratory tract infections lacks specificity (17). As a result, molecular diagnosis has become the gold standard for accurate identification.

Various molecular assays and technologies are available for respiratory viral testing. These include single-target tests, panels for SARS-CoV-2, influenza, and RSV, as well as extended panels that multiplex testing for 19 or more respiratory viral and bacterial targets. Current recommendations favor testing, primarily when results inform patient management or infection control decisions (2). However, testing decisions are influenced by multiple factors, including access, cost, patient symptoms, immune status, healthcare settings, laboratory complexity, and available test options. Since the onset of the COVID-19 pandemic, testing for SARS-CoV-2 has surpassed testing for all other respiratory viral pathogens. During respiratory virus season, testing volumes for SARS-CoV-2, influenza, and RSV increase. Testing for other respiratory viral pathogens is less common and generally limited to immunocompromised, hospitalized, and pediatric patients (1).

We previously showed that respiratory viruses other than influenza, SARS-CoV-2, and RSV that circulate at high rates (rhinovirus/enterovirus) but are not as frequently tested can be associated with high morbidity and hospital admissions (1). Given the cost of extended respiratory panels, testing options that could offer flexibility in customizing panel targets based on local and institutional prevalence and clinical needs can help increase the diagnostic yield and reduce the associated cost. In this study, we evaluated the LIAISON PLEX Respiratory *Flex* Assay, a recent FDA-cleared extended respiratory syndromic test that allows for the selection of customized panels. The assay tests for all targets; however, results are interpreted only for ordered or custom panel targets. If additional targets are added to an order, there is no need to run new samples—ordered targets will be interpreted using analysis of archived runs. Given the pioneer features of this panel, we wanted to evaluate, using Johns Hopkins Health System (JHHS) data, what pathogens can be considered for testing as a primary tier to maximally increase the diagnostic likelihood at a similar cost to SARS-CoV-2/Flu/RSV panels.

A cohort of 200 patients who presented with symptoms and were tested negative for SARS-CoV-2/Flu/RSV were tested with the LIAISON PLEX Respiratory *Flex* Assay. The time frame of testing was between December 2023 and September 2024 to capture different respiratory viral seasons and prevalence. The total positivity rate of the LIAISON PLEX Respiratory *Flex* Assay for this cohort was 31%, and the most commonly detected pathogens were rhinovirus/enterovirus, adenovirus, coronavirus, and HPIV3. During the study time frame, a total of 18,373 patients presented at JHHS with symptoms and tested negative for SARS-CoV-2/Flu/RSV. Weighted estimates of positivity using our tested cohort indicated that 14.3% would have tested positive for rhinovirus/enterovirus, followed by HPIV3 at 2.7%, adenovirus at 2.6%, and coronavirus at 2.1%. Patients’ results and weighted total estimates were consistent with the JHHS and Maryland State positivity rates. The results indicate that using a panel that adds rhinovirus/enterovirus to influenza, SARS-CoV-2, and RSV panels will increase the diagnostic yield from 12.5% when only using SARS-CoV-2/Flu/RSV to close to 26%. The data also indicate that institutional and local positivity rates can guide target selection for custom panels and emphasize the value of considering adding testing for the year-long circulating rhinovirus/enterovirus and adenovirus targets.

Our results showed that of the 18,373 patients who had an initial order for SARS-CoV-2/Flu/RSV, 1,109 (6%) received an order for the standard of care ePlex RP.2 in the same encounter. If the Flex feature of the LIAISON PLEX were leveraged to avoid a second swab collection (in institutions that currently collect a second swab for sample source—nasal vs nasopharyngeal—or compliance reasons), an estimated 3,327 minutes of staff time for sample collection and 2,218 minutes for running the ePlex RP.2 could be saved (during our study time frame) (4), along with cost savings in reagents and avoidance of duplicate target testing. Diagnosing viral causes of pneumonia and other mild to severe respiratory infections is also expected to enhance antibiotics stewardship and reduce the unnecessary use of antibiotics (in our cohort, 1%).

In conclusion, the LIAISON PLEX Respiratory *Flex* Assay has comparable analytical and clinical performance to the JHHS standard of care ePlex RP.2. The flexibility in designing respiratory viral (and bacterial) testing tiers is expected to save staff time and reagent costs while increasing the clinical diagnostic yield. A timely example is the *Mycoplasma pneumonia*e outbreak. With such unexpected outbreaks, a quick adjustment of diagnostic panels is an attractive approach. The LIAISON *Flex’s* ability to only analyze and interpret data when additional targets are ordered is an optimal solution for ethical and compliance considerations, and the flexibility of customizing panels can have a significant clinical and financial impact. However, the clinical implementation of such an approach can be challenging and requires extensive validations. In addition, waived and decentralized solutions for testing SARS-CoV-2/Flu/RSV in the emergency departments facilitate rapid disposition. Our study was limited in size, and hence, we were not powered to evaluate the impact of patient populations on selected targets (e.g., age and immune status). Our clinical outcome analysis was also limited by the retrospective nature of the study. Future studies will reveal the clinical significance of accurately and promptly diagnosing respiratory viral infections and define flexible approaches for ordering, modifying, and reimbursing custom panels.

## SUPPLEMENTAL MATERIAL

Table S1 jcm.00313-25-s0001.docx Concordance of the LIAISON PLEX Respiratory *Flex* Assay and the standard of care ePlex RP2 panel.

ASM does not own the copyrights to Supplemental Material that may be linked to, or accessed through, an article. The authors have granted ASM a non-exclusive, world-wide license to publish the Supplemental Material files. Please contact the corresponding author directly for reuse.
