# Supplementary material for: The utility of syndromic respiratory pathogen panels: the premise of flexible and customizable approaches
Source: J Clin Microbiol. 2025 Jun 10;63(7):e00313-25. doi: 10.1128/jcm.00313-25 (PMC12239722; doi:10.1128/jcm.00313-25)
Supplement: Table S1 — Concordance of the LIAISON PLEX Respiratory Flex Assay and the standard of care ePlex RP2 panel. [file jcm.00313-25-s0001.docx]

| Sample ID | **ePlex RP2 (standard of care)** | **LIAISON PLEX**^®^ **Respiratory *Flex*** | **Discordant analysis (BioFire RP.2)** |
| --- | --- | --- | --- |
| 140 | Adenovirus | Adenovirus (A / B / C / D / E / F) Enterovirus/Rhinovirus | Adenovirus |
| 139 | Coronavirus | Coronavirus (HKU1/229E/NL63/OC43) | Coronavirus 229E |
| 124 | Human metapneumovirus | Negative | Human metapneumovirus |
|  |  |  |  |
| 136 | Influenza B | Hybridization Deck Failure No Call | Influenza B |
| 200 | Negative | *Mycoplasma pneumoniae* | *Mycoplasma pneumoniae* |
| 155 | Coronavirus | Negative | Negative |
| 25 | Human parainfluenza virus 2 | Human parainfluenza virus 2 Coronavirus (HKU1/229E/NL63/OC43) | Negative |
| 120 | Human parainfluenza virus 4 | Negative | Negative |
| 143 | SARS-CoV-2 | Negative | SARS-CoV-2 |
| 154 | SARS-CoV-2 | Negative | SARS-CoV-2 |
| 142 | SARS-CoV-2 | SARS-CoV-2 Enterovirus/rhinovirus | SARS-CoV-2 Enterovirus/rhinovirus |
| 121 | Human parainfluenza virus 4 | Human parainfluenza virus 4 | Human parainfluenza virus 4 |
| 141 | Adenovirus | Adenovirus (A / B / C / D / E / F) |  |
| 129 | Adenovirus | Adenovirus (A / B / C / D / E / F) |  |
| 317 | *Chlamydia pneumoniae Mycoplasma pneumoniae* | *Chlamydia pneumoniae Mycoplasma pneumoniae* |  |
| 131 | Coronavirus | Coronavirus (HKU1/229E/NL63/OC43) |  |
| 123 | Coronavirus | Coronavirus (HKU1/229E/NL63/OC43) |  |
| 146 | SARS-CoV-2 | SARS-CoV-2 |  |
| 153 | SARS-CoV-2 | SARS-CoV-2 |  |
| 149 | Influenza A | Influenza A (subtype H1) Influenza A |  |
| 147 | Influenza A | Influenza A (subtype H3) Influenza A |  |
| 148 | Influenza A | Influenza A (subtype H1) Influenza A |  |
| 150 | Influenza B | Influenza B |  |
| 137 | Influenza B | Influenza B |  |
| 138 | Influenza B | Influenza B |  |
| 110 | Human metapneumovirus | Human metapneumovirus |  |
| 117 | Human metapneumovirus | Human metapneumovirus |  |
| 152 | Human metapneumovirus | Human metapneumovirus |  |
| 133 | *Mycoplasma pneumoniae* | *Mycoplasma pneumoniae* |  |
| 128 | *Mycoplasma pneumoniae* | *Mycoplasma pneumoniae* |  |
| 127 | *Mycoplasma pneumoniae* | *Mycoplasma pneumoniae* |  |
| 20 | Human parainfluenza virus 1 | Human parainfluenza virus 1 |  |
| 21 | Human parainfluenza virus 1 | Human parainfluenza virus 1 |  |
| 132 | Human parainfluenza virus 1 | Human parainfluenza virus 1 |  |
| 22 | Human parainfluenza virus 2 | Human parainfluenza virus 2 |  |
| 23 | Human parainfluenza virus 2 | Human parainfluenza virus 2 |  |
| 24 | Human parainfluenza virus 2 | Human parainfluenza virus 2 |  |
| 116 | Human parainfluenza virus 3 | Human parainfluenza virus 3 |  |
| 118 | Human parainfluenza virus 3 | Human parainfluenza virus 3 |  |
| 119 | Human parainfluenza virus 3 | Human parainfluenza virus 3 |  |
| 151 | Human parainfluenza virus 4 | Human parainfluenza virus 4 |  |
| 122 | Human parainfluenza virus 4 | Human parainfluenza virus 4 |  |
| 111 | Enterovirus/rhinovirus | Enterovirus/rhinovirus |  |
| 126 | Enterovirus/rhinovirus | Enterovirus/rhinovirus |  |
| 125 | Enterovirus/rhinovirus | Enterovirus/rhinovirus |  |
| 156 | Respiratory syncytial virus A | Respiratory Syncytial Virus (RSV A and RSV B) |  |
| 157 | Respiratory syncytial virus A | Respiratory Syncytial Virus (RSV A and RSV B) |  |
| 144 | Respiratory syncytial virus A | Respiratory Syncytial Virus (RSV A and RSV B) |  |
| 145 | Respiratory syncytial virus A | Respiratory Syncytial Virus (RSV A and RSV B) |  |
| 135 | Respiratory syncytial virus B | Respiratory Syncytial Virus (RSV A and RSV B) |  |
| 134 | Respiratory syncytial virus B | Respiratory Syncytial Virus (RSV A and RSV B) |  |
| 130 | Respiratory syncytial virus B | Respiratory Syncytial Virus (RSV A and RSV B) |  |

Table S1. Concordance of the LIAISON PLEX^®^ Respiratory *Flex* Assay and the standard of care ePlex RP2 panel.
